# Investigating Granulovacuolar Degeneration in Alzheimer’s Disease and Other Tauopathy Models

**DOI:** 10.1002/alz.095613

**Published:** 2025-01-09

**Authors:** Daniel S Dai, Tak Ian Chio, Virginia M.‐Y. Lee

**Affiliations:** ^1^ University of Pennsylvania, Philadelphia, PA USA; ^2^ University of Pennsylvania, College of Arts and Sciences, Philadelphia, PA USA; ^3^ Center for Neurodegenerative Disease Research, Department of Pathology and Laboratory Medicine, University of Pennsylvania, Perelman School of Medicine, Philadelphia, PA USA; ^4^ Institute on Aging, University of Pennsylvania, Perelman School of Medicine, Philadelphia, PA USA

## Abstract

**Background:**

Granulovacuolar degeneration (GVD) is a common feature in Alzheimer’s disease (AD) and other tauopathies such as corticobasal degeneration (CBD), progressive supranuclear palsy (PSP), and Pick’s disease (PiD). Despite its prevalence, much remains unknown about the role of GVD in the pathobiology of these diseases. Morphologically, GVD is characterized by intraneuronal membrane‐bound vacuoles, also known as GVD bodies (GVBs). Previously, it has been observed that *in vitro* and *in vivo* mouse models of tauopathy can also induce GVB formation. Nevertheless, the *in vivo* models in particular remain poorly characterized. Herein, we provide a more systematic characterization of GVD in the *in vivo* mouse models to better enable elucidation of GVD and its relation to tau pathology and neurodegeneration.

**Method:**

Brain sections were retrieved from various tauopathy mouse models. GVB distribution was characterized via immunohistochemistry using CK1δ as the primary GVB marker. Co‐staining with a phospho‐tau antibody (AT8) confirmed the presence of tau pathology in all of our models.

**Results:**

CK1δ+ GVBs were detected in multiple tauopathy mouse models, including PS19 as well as AD‐tau‐injected wild type, 5xFAD, and 6hTau mice. The level of GVBs differ between mouse models and correlate with the level of tau pathology that was developed in these mice. In particular for the 6hTau mice, where the amount of GVBs was the most robust, temporal characterization of GVB formation shows the most significant increase in the number of GVB+ neurons from 1 to 3 months post‐AD‐tau injection. At the single cell level, the number of CK1δ+ puncta also increased over time. By 3 mpi, GVBs were detected beyond the hippocampus (site of injection) and in the cortical regions where tau pathology was also present. Finally, 6hTau mice injected with CBD‐, PSP‐, and PiD‐tau all developed GVBs, albeit at variable levels that parallel the extent of tau pathology.

**Conclusions:**

GVB development tracks with tau pathology development in our *in vivo* mouse models. The increase in GVBs within a cell over time suggests that GVBs continue to form as tau pathology matures. This induction of GVBs by tau pathology is irrespective of the different tau strains from different tauopathies.

